# When Ultrasonic Sensors and Computer Vision Join Forces for Efficient Obstacle Detection and Recognition

**DOI:** 10.3390/s16111807

**Published:** 2016-10-28

**Authors:** Bogdan Mocanu, Ruxandra Tapu, Titus Zaharia

**Affiliations:** 1 ARTEMIS Department, Institut Mines-Télécom/Télécom SudParis, UMR CNRS MAP5 8145, 9 rue Charles Fourier, Évry 91000, France; bogdan.mocanu@telecom-sudparis.eu (B.M.); titus.zaharia@telecom-sudparis.eu (T.Z.); 2 Telecommunication Department, Faculty of ETTI, University Politehnica of Bucharest, Splaiul Independentei 313, Bucharest 060042, Romania

**Keywords:** wearable assistive device, obstacle detection, object recognition, acoustic feedback, ultrasonic network, computer vision techniques, machine learning algorithms

## Abstract

In the most recent report published by the World Health Organization concerning people with visual disabilities it is highlighted that by the year 2020, worldwide, the number of completely blind people will reach 75 million, while the number of visually impaired (VI) people will rise to 250 million. Within this context, the development of dedicated electronic travel aid (ETA) systems, able to increase the safe displacement of VI people in indoor/outdoor spaces, while providing additional cognition of the environment becomes of outmost importance. This paper introduces a novel wearable assistive device designed to facilitate the autonomous navigation of blind and VI people in highly dynamic urban scenes. The system exploits two independent sources of information: ultrasonic sensors and the video camera embedded in a regular smartphone. The underlying methodology exploits computer vision and machine learning techniques and makes it possible to identify accurately both static and highly dynamic objects existent in a scene, regardless on their location, size or shape. In addition, the proposed system is able to acquire information about the environment, semantically interpret it and alert users about possible dangerous situations through acoustic feedback. To determine the performance of the proposed methodology we have performed an extensive objective and subjective experimental evaluation with the help of 21 VI subjects from two blind associations. The users pointed out that our prototype is highly helpful in increasing the mobility, while being friendly and easy to learn.

## 1. Introduction

The report published by the World Health Organization (WHO) [1] in August 2014 about people with visual disabilities points out that over 285 million people worldwide are estimated to suffer from visually impairments (VI). Among this population, 39 million are completely blind while 246 million people have low vision. Approximately 90% of VI people are living in developing countries but have a low income. Moreover, the WHO has predicted that by the year 2020 the number of blind (resp. partially sighted) people will reach 75 million (resp. 250 million). For VI people, common activities naturally performed by normal humans (such as safe navigation in indoor/outdoor environment, shopping or familiar face recognition) are highly difficult tasks. One of the most challenging activity concerns the autonomous displacement in an unknown environment because of its high degree of danger (i.e., possible collision with static or dynamic obstacles). For this reason, most VI people walk on known routes while continuously memorizing novel elements.

In order to acquire additional information about the near surrounding, VI people rely on traditional assistive elements such as the white cane or guide dogs [2]. However, such approaches show quickly their limitations when confronted with real life scenarios. The white cane is designed to detect possible obstructions situated in the near vicinity of the user, but requires a physical contact with the object. The white cane also serves to another important purpose: to alert normal people about the presence of a blind/VI person. In such way, they can protect them by moving out from their walking path. Even so, the white cane cannot determine an object’s degree of danger or detect obstructions located at the head level. On the other hand, guide dogs are considered a more effective solution, but are very expensive, the have a reduced operational time and require an extensive training phase. In this context, developing an assistive technology that can interpret in real-time the urban scenes and provide to the blind and visually impaired users with fine cognition of the environment, becomes a crucial challenge.

In this paper, we propose a novel navigation assistant that facilitates the safe movement of VI people in urban scenes by using both sensors and computer vision technologies. The system is able to acquire information about the environment, interpret it and alert users about possible dangerous situations/obstacles. At the hardware level, the proposed framework is composed of a regular smartphone device, Bluetooth bone conduction headphones, ultrasonic sensors, an Arduino microcontroller and a flexible waist belt. Our solution can be characterized as low-cost and easy to wear because it is based on general public components available on the market, and does not require potentially costly, dedicated components. The major contribution of the paper concerns the fusion of two independent information sources, including ultrasonic sensors and the embedded video camera, which makes it possible to obtain a wearable assistive device that can reliably function in both outdoor and indoor scenarios. The framework is designed to detect and recognize, in real-time, both static and dynamic obstacles using a smartphone device as a processing unit. At the software level our major contribution concerns the effective integration, optimization and adaptation of different machine vision/learning techniques, while fully taking into account the information provided by ultrasonic module. Our system is carefully conceived to transmit warning messages fast enough for a VI user who is walking normally.

The rest of the paper is organized as follows: Section 2 reviews the state of the art in the areas of VI-dedicated assistive technologies. We have focused our attention on Electronic Travel Aid (ETA) technologies based on both sensors and computer vision methods. Section 3 introduces first the proposed obstacle detection framework, and then details the object recognition methodology. Section 4 describes the experimental results obtained and includes both an objective evaluation, related to the obstacle detection/recognition performances and a subjective evaluation performed with the help of VI users in real life urban scenes. Finally, Section 5 concludes the paper and presents some perspectives of future work.

## 2. Related Work

In the last years various Electronic Travel Aid (ETA) technologies have been introduced. They were developed as a digital enhancement to the traditional white cane and aim at increasing the mobility of VI users. The following sub-sections describe and analyze the state of the art in the field, with systems based either on sensor networks or computer vision techniques.

### 2.1. Sensor-Based ETA

The sensor-based ETA systems are designed to collect environmental information and to transmit it to the VI user through a set of acoustic or haptic signals. 

One of the first ETA introduced in the state of the art was based on Mowat sensors [3], which emit a high frequency sound to detect objects (and calculate the distance to the object), within a narrow beam. The Mowat system is a hand-held device that informs the VI user about the distance between the detected objects by means of tactile vibrations. The frequency of the vibrations is inversely proportional to the distance between the sensor and the object. The experimental evaluation performed with VI users shows that the Mowat device is helpful and able to facilitate the autonomous navigation. However, the hands-free condition required by VI people is violated. In addition, effectively using the system requires an extensive training phase. Today, Mowat devices are no longer in production.

In [4], two sonar-based environmental imaging sensors, called Sonicguide and Trisensor are proposed, which provide spatial information about the detected obstacles. The Sonicguide is a head-mounted binaural device that uses ultrasonic echo location. The system is today commercially available on the market. The Trisensor device consists of a triad of high-resolution ultrasonic spatial sensors integrated on a head-mounted device. The three sensors cover a 50° forward field of view and create an auditory image through stereo headphones. The Trisensor allows the detection and location of multiple obstacles in the 3D space up to 5 m in front of the user.

The binaural sonar introduced in [5] detects objects situated at arbitrary locations. The sonar employs a pair of Polaroid 6500 ranging modules connected to Polaroid 7000 transducers operating simultaneously in a binaural array configuration. The system is able to reliably determine if the object lies on the left or on the right side of the sonar axis. The warning messages are transmitted to VI users through vibrotactile stimulation.

The GuideCane approach that also uses an ultrasonic sensor to detect obstacles and an embedded computer to determine the optimal walking path was proposed in [6]. The navigation directions are sent to the user as a tactile stimulation felt in the cane handle. The framework is operational in real-time. However, the system cannot provide navigation information and is unable to detect overhanging obstacles or borders of a sidewalk.

The EPFL system proposed in [7] uses a multi-sonar architecture to provide obstacle avoidance abilities. The warning messages are sent using vibro-tactile actuators. The framework was tested solely in indoor and simulated scenarios. The blindfolded user is able to walk normally, through corridors, without any collision with obstacles situated at various levels of height. However, the system returns a prohibitive number of false alarms and cannot be adopted in outdoor scenes.

In [8], the CyARM system is designed to provide users with an intuitive perception of the living space. The system is mounted on the users’ body through wires and estimates the distances between VI and obstacles using ultrasonic waves. The measured distance is translated into different wire tensions. The framework has been extensively evaluated and returns a high detection rate, while estimating with high accuracy the real distances. However, when facing dynamic obstacles the overall performance decreases significantly (with more than 30% false detections).

The SUGAR system, recently introduced in [9], is an indoor navigation device for visually impaired people, which uses a smartphone as a processing unit and Ultra-wideband (UWB or also known as digital pulse wireless) technology for positioning. Ultra-wideband is a wireless technology for transmitting large amounts of digital data over a wide spectrum of frequency bands with very low power for a short distance. In the context of VI-dedicated applications, the UWB technology is encountered in radar systems, in which the signal penetrates nearby surfaces, but reflects surfaces that are further away, thus allowing objects to be detected behind walls or other coverings. The warning messages are transmitted to the VI users through acoustic signals and voice commands played on headphones. The experimental evaluation performed with blind persons validates the proposed approach. However, the framework is limited to indoor application and to environments containing Ubisense UWB tags. By using regular headphones the user ears are always blocked and cannot infer sounds from the environment. Nothing is said about the power consumption or the system lifetime. The ETA proposed in [10] is based on a multi-section sensing in order to reliably detect obstacles. The user feedback is transmitted through vibrotactile stimulation. The system can be regarded as an electronic cane identifying obstacles situated at arbitrary levels of height. Compared with other ETA as [7] or [8], the system has a reduced cost and does not require an extensive training phase.

The analysis of the state of the art shows that the assistive devices based on sensorial substitution of human vision can be very effective when used in indoor environments or when employed to detect large, flat structures. However, in the case of outdoor urban scenarios the systems’ sensitivity becomes prohibitively high. Moreover, ETAs based on sonar are sometimes unable to identify with high confidence dynamic objects situated in the near vicinity of the user or other important features such as the borders of a sidewalk and stairs. Regarding the hardware architecture, the frameworks recently proposed in [9,10] can be characterized as lightweight and comfortable to wear. Still, they require an important training phase and an adequate user interface. Even so, the information captured by the human eyes cannot be completely substituted by other senses such as acoustic or tactile. The computer vision techniques notably aim at solving such problems, and offer a higher level of understanding, reproduction and interpretation of real urban scenes.

### 2.2. ETA Using Computer Vision Techniques

Recent advances in computer vision techniques have led to the rapid proliferation of assistive devices based on artificial intelligence, dedicated to outdoor/indoor navigation. In [11], the SmartVision prototype is designed to provide navigation information and detect sidewalk boundaries and obstacles located in front of the user, beyond the area reached by the white cane. The system is composed of a stereo camera, a portable computer and one earphone speaker. The system is sensitive to the GPS signal strength. Moreover, the performances can be severely impacted by the accidental leaving of the correct walking path or by the presence of multiple types of edges in the scene (e.g., street intersection or crossroads). A real-time obstacle detection system for VI people that uses a handheld smartphone is proposed in [12]. The authors apply traditional computer vision techniques, including color histogram representation and edge detection to identify on-floor obstacles in real-time, without any a priori training. Even though the system is non-intrusive and cheap, it violates the hand free condition imposed by VI users. Moreover, it cannot detect overhanging obstructions and no evaluation is performed in real, outdoor scenarios.

In our previous work presented in [13], a complete framework for obstacle detection and classification integrated on a regular Smartphone device was proposed. The system was tested in outdoor scenarios with actual VI users and returned high precision and recall scores. However, the framework cannot identify large structures as walls, doors or obstruction with the size greater than half of the image length. In addition, the system is unable to correctly estimate the distance between the VI user and an obstruction and is influenced by the user’s own movement.

A six degrees of freedom (6DOF) simultaneous localization and mapping (SLAM) solution embedded on a wearable device and using two stereo cameras is proposed in [14]. The system works nearly in real-time and can determine the position of the VI user by applying ego-motion estimation. Both 3D and 2D information is integrated into a global rectification algorithm that supports 6DOF. The authors point out that the proposed algorithm yields solely a metric map and becomes useless for a blind people unless some additional semantic information is incorporated.

A navigation assistant using a head-mounted stereo-vision system is proposed in [15]. By combining the camera pose information with the dense 3D data obtained from the stereo triangulation, a vicinity map of the user environment is constructed. A safe walking path (obstacle free) is determined, while the warning messages are transmitted through micro-vibration stimulation. However, the system requires a powerful processing unit and is considered invasive.

In [16], the proposed aerial obstacle detection framework is based on a 3D smartphone that captures the 3D structure of the scene through stereo vision. With the help of magnetometer and accelerometer sensors, the system establishes the walking direction of a VI user by identifying potential dangerous obstacles along the path. The system has been tested with actual blind users and proved to be effective. However, the approach suffers from its sensitivity to any sudden camera movement and changes in the lighting conditions. In such cases, the tracking process cannot be achieved successfully.

With the developments of the Red, Green, Blue and Depth (RGB-D) cameras, different guidance systems [17,18,19], have been developed. In [17], an assistive device based on depth information was proposed. The framework is designed to detect multiple dynamic obstacles and transmit this information to users in order to ensure safe navigation. The efficiency and robustness of the approach were measured with the help of VI people in real scenarios. As pointed out by the authors, the system has a relatively reduced applicability (only for indoor spaces). Moreover, the use of regular headphones is inappropriate in the context of VI-dedicated applications because the warning messages can interfere with other sounds from the environment.

An assistive device designed to recognize 3D objects is proposed in [18]. At the hardware level, the framework is based on a Microsoft Kinect sensor, a controller, a tactile device and a processing unit. The vibration feedback is transmitted only for objects situated in front of the users (in the Kinect field of view). Similarly, in [19] the authors introduce a system that uses the acoustic signals to convey obstacle information acquired from Kinect sensors. Both frameworks proposed in [18,19] are sensitive to the object recognition training phase and can be used only in indoor environment. A vision-based SLAM approach is used in [20] in order to detect and track independently moving objects. The proposed system can estimate the camera egomotion as well as different types of movement in the scene, and is able to perform a 3D map reconstruction. The entire framework can function in real-time on a low-end CPU. However, in an outdoor space the system is highly sensitive to changes in the illumination conditions, which affects the 3D reconstruction of the environment and the estimation of the depth maps. Moreover, the development of the 3D scene model requires an extensive processing power. When integrated on a smartphone device with a regular Advanced Reduced instruction set computing Machine (ARM) architecture, the method is not able to perform in real-time, even when applying the optimizations proposed by authors.

Table 1 summarizes the various VI-dedicated assistive devices presented in this section and proposes a qualitative comparison. The considered evaluation criteria concern the following set of features: cost (the system has to be affordable to most users), capacity to function in real-time, portability (the system has to be light, small and ergonomic), wearable capacity, reliability (robustness with respect to the outdoor dynamics) and friendliness (easy to learn without an important training stage).

The analysis of the state of the art shows that, until now, no method can fulfill to a satisfactory degree all the features required to facilitate the safe movement of VI people in an unknown outdoor environment. When comparing the sensor-based systems with frameworks relying on computer vision it can be observed that each approach presents advantages and limitations, but no single method is robust enough to replace the white cane. Starting from this observation, in this paper we propose a novel system that uses two sources of information from both ultrasonic sensors and a regular 2D video camera in order to acquire sufficient data for a comprehensive understanding of the scene. The proposed approach is described in the following section.

## 3. Hybrid Computer Vision and Ultrasonic Sensors Approach

**Figure 1:**
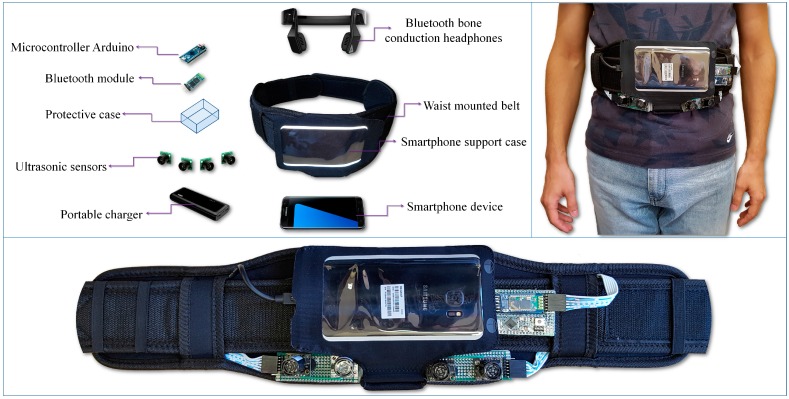
The hardware architecture of the proposed framework.

The hardware components considered and integrated in the proposed device (Figure 1) include: a regular smartphone device (Samsung S7, Samsung Electronics Co., Suwon, South Korea), Bluetooth bone conducting headphones (AfterShokz Bluez 2, Voxlink LLC, East Syracuse Onondaga, New York, NY, USA), a microcontroller (Arduino Micro, Arduino LLC, Somerville, MA, USA), a bluetooth device (Master Slave HC-05, Wavesen, Guangzhou, China), four ultrasonic sensors (MaxSonar LV EZ-0, MaxBotix Inc., Brainerd, MN, USA), an external battery (Anker Astro E5 16,000 mAh with a weight of 308 grams, Anker Technology Co., Mongkok Kl, Honk Kong) and a smartphone waist belt.

**Figure 2:**
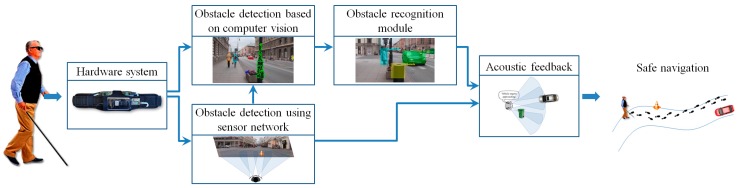
The software architecture of the proposed framework.

In order to facilitate the safe movement of the VI people in an outdoor/indoor environment, at the software level, we introduce two novel modules able to detect and recognize possible dangerous static or dynamic obstacles existent on the walking path (Figure 2). The obstacle detection module is based on a fusion of information acquired from the smartphone video camera and from the ultrasonic sensors. Then, the detected obstacles are applied as input to the recognition module that semantically understand and interpret the scene. Depending on the obstructions position and their relative degree of danger, prioritized acoustic signals are transmitted to the VI user.

Let us now present the proposed obstacle detection module, which jointly exploits the video signal acquired by the smartphone camera and the information provided by the ultrasonic sensor, within the framework of an interest-point object detection approach, exploiting apparent motion analysis and integrating distance information.

### 3.1. Obstacle Detection Framework

Let us first detail how the video signal is exploited for both static and dynamic obstacle detection purposes.

#### 3.1.1. Detection of Static/Dynamic Obstacles Using Computer Vision Techniques

##### Confident Points of Interest Extraction

**Figure 3:**
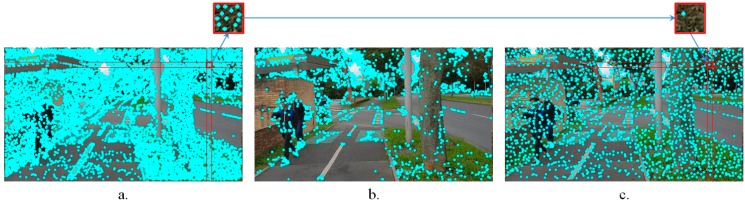
Confident point of interest selection using: (**a**) The traditional FAST algorithm; (**b**) The method introduced in [23]; (**c**) The proposed filtering strategy.

The method starts by analyzing the first frame captured by the video camera of the smartphone device. We have evaluated several different point of interest extraction algorithms, including SIFT [21], SURF [22], BRIEF [23] or FAST [24]. For highly textured areas, often associated with background regions, the number of retained points of interest becomes prohibitively high (Figure 3a). Because the objective is to develop a real-time application, we have decided to use the FAST approach, which presents the lowest computation time. Furthermore, the total number of points of interest is reduced by applying a simple, yet effective filtering strategy.

To this purpose, a regular rectangular grid, overlapped on the video frame is constructed. The points of interest associated to each cell are then determined. For each cell, solely the most relevant points, i.e., those which present the highest value of the Harris Laplacian operator [25] are retained. Compared to other filtering strategies such as the one introduced in [26] that selects the most important top-*k* candidate points from the entire image, without considering the spatial information (Figure 3b), the proposed method ensures that the retained points are more evenly distributed within the image. In the same time, the approach makes it possible to avoid dense, agglomerate points of interest clusters that can appear in certain areas (Figure 3c). Let us underline that in Figure 3b,c the same number of points of interest is retained.

The performance of the point of interest selection strategy is controlled by the size of the grid cell defined as: $Grid_{cell}=\left(I_{W}\cdot I_{H}\right)/N_{points}$, where $I_{H}$ and $I_{W}$ represent the image height and width, while $N_{points}$ is the total number of points retained for an image. In the experiments, for a video stream with a resolution of 320 × 240 pixels, the value $N_{points}$ has been set to 1000 points.

##### Point of Interest Tracking

In order to track the representative points of interest between successive frames, the multi-resolution Lucas-Kanade (LK) algorithm [27] has been adopted, which offers a fair compromise between the accuracy of the estimated motion vectors and the processing speed. We have applied as input to the LK method the set of relevant points of interest obtained using the strategy described above and tracked them through the video stream. The tracker is locally reinitialized whenever such an action is required (i.e., for disappearing objects, for new regions entering in the camera field of view or for blurred areas where the tracking cannot be performed). The LK algorithm determines, for each point of interest $\;p_{1i}\left(x_{1i},\;y_{1i}\right)$ its corresponding one in the successive frame $p_{2i}\left(x_{2i},\;y_{2i}\right)$, as well as the associated motion vector $v_{i}\left(v_{ix},\;v_{iy}\right)$, expressed in terms of magnitude and orientation:

(1) $$M_{12}=\sqrt{v_{ix}^{2}+v_{iy}^{2}};\alpha_{12}=arctg\frac{v_{iy}}{v_{ix}}+k\pi \;,\;k\in \mathbb{Z}$$

where $v_{ix}$ and $v_{iy}$ respectively represent the horizontal and vertical velocities.

##### Background/Camera Movement Estimation

The scene global motion is estimated using the RANSAC algorithm [28] that determines the optimal homographic transformation **H** between two successive video frames. For each relevant interest point $p_{1i}{\left(x_{1i},\;y_{1i},1\right)}^{T},$ expressed in homogeneous coordinates, its new position $p_{2i}^{est}{\left(x_{1i}^{est},\;y_{1i}^{est},1\right)}^{T}$ is computed as described in the following equation:

(2) $$\left[\begin{array}{c} \begin{array}{c} x_{1i}^{est}\\ y_{1i}^{est} \end{array}\\ w \end{array}\right]=\left[\begin{array}{c} h_{11}\;h_{12}\;h_{13}\\ h_{21}\;h_{22}\;h_{23}\\ h_{31}\;h_{32}\;h_{33}\; \end{array}\right]\cdot \left[\begin{array}{c} \begin{array}{c} x_{1i}\\ y_{1i} \end{array}\\ 1 \end{array}\right];$$

where $w=1/\left(h_{31}\cdot x_{1i}+h_{32}\cdot y_{1i}+h_{33}\right)$. The error ($E_{r}$) between the estimated position $p_{2i}^{est}$ and the actual location ($p_{2i}$) of the interest point is evaluated using the Euclidian distance as:

(3) $$E_{r}\left(p_{2i}^{est}\right)=||p_{2i}^{est}-p_{2i}||,$$

where ||·|| denotes the *L_2_* norm. In the ideal case, the estimation error ($E_{r}$) should be zero which implies that all points positions can be correctly determined by using the homographic matrix **H**. However, in real life applications some degree of error is inevitable and $E_{r}$ needs to be compared to a threshold $Th_{1}$ in order to determine the set of points (called *inliers*) that satisfy the transformation. The remaining points are considered as *outliers* and correspond to foreground objects.

The user displacement affects significantly the video acquisition process, even in the case where the smartphone is attached on a waist belt. The video stream is unstable and presents cycling pan, tilt oscillation or panoramic distortions.

**Figure 4:**
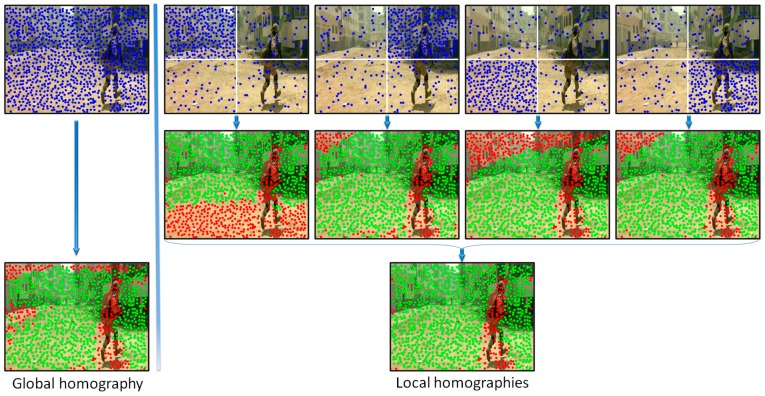
Background motion estimation using global versus local homographies (green points correspond to the background, while the red points are labeled as belonging to foreground objects in apparent motion).

In order to increase the robustness of the global motion estimation model we have proposed to analyze distinctively various areas of the frame. Multiple homographies have been computed over the same image by using different sets of points of interest. More precisely, the image is divided into four equal, non-overlapping rectangles (Figure 4). Then, four independent homographies are computed, one for each rectangle. For a given rectangle, a homographic matrix (**H**) is determined by using all the points of interest included in the current rectangle and only 25% of the points of interest from other regions, randomly selected. Next, each homographic matrix is used to determine its set of *inliers/outliers*. Finally, the interest points are labeled as belonging to an object motion if the estimation error is superior to a threshold $Th_{1}$ in all four homographies.

Figure 4 presents two different ways of estimating the camera motion: (a) when using all the points of interest as input to the RANSAC algorithm (i.e., global homography); (b) when applying the proposed strategy based on local homographies. The points marked with green are assigned to the background/camera motion, while the red points define other types of motion existent in the scene. We can observe that the local approach makes it possible to distinguish in a more reliable manner between foreground and background points of interest. The set of *outlier* points are further used to recognize different types of objects existent in the scene.

##### Foreground Object Detection

Because of the camera motion induced by the user’s movement, even static foreground objects present an apparent motion that can help distinguish them from the background. In addition, various dynamic obstacles, including vehicles, bicycles or pedestrians can be identified by performing the same motion analysis process, detailed in the following paragraphs.

**Figure 5:**
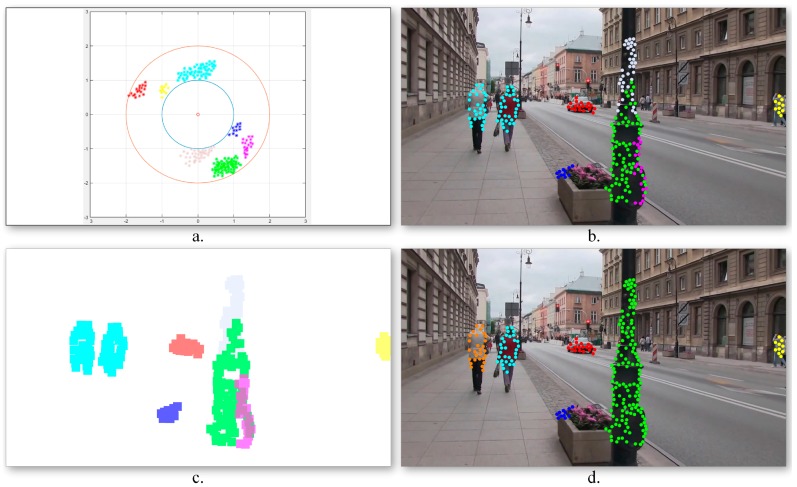
Foreground motion estimation: (**a**) Points of interest distribution in the Cartesian space constrained to lay on an annulus; (**b**) Motion class estimation using *k*-means clustering; (**c**) Motion cluster binary masks; (**d**) Cluster division/merging based on spatial constraints.

A clustering algorithm that uses as input the points of interest with the associated motion vectors is first applied. However, it is not feasible to use directly the motion vectors’ magnitude and orientation expressed in polar coordinates because the angular coordinate has a circular range of values, varying from 0 to 2 π. Most of the clustering techniques assume that the data is distributed in a vector space and compute the *L_2_* distance between different samples in order to form groups. Based on this observation, the proposed algorithm integrates the following non-linear transform that changes the motion vectors parameters from polar coordinates back to the 2D Cartesian space:

(4) $$v_{ix}^{\prime }=d\cdot \cos\alpha_{12};\;v_{iy}^{\prime }=d\cdot \sin\alpha_{12},$$

where $\alpha_{12}$ represents the point of interest motion vector orientation and *d* is the radial coordinate that incorporates the magnitude information. The value of d is computed as:

(5) $$d=1+\frac{M_{12}}{M_{max}},$$

where $M_{max}$ is the maximum magnitude value for all motion vectors from the current frame. In this manner, all the motion vectors are constrained to lay on an annular domain defined by two circles with radii equal to 1 and 2, respectively. The transform allows interpreting angles of 0 and 2 π as being equivalent while distinguishing between different positions. Figure 5a illustrates the obtained motion vector distribution.

Next, in order to identify the various moving objects, a *k*-means algorithm is applied. The algorithm aims at partitioning the *outlier* points of interest into a set of groups $S=\left\{S_{1},S_{2},\ldots ,S_{k}\right\}$ that minimizes the following intra-cluster mean square distance:

(6) $$\arg\underset{S}{\min}\overset{k}{\underset{j=1}{\sum }}\underset{v_{i}^{\prime }\in S}{\sum }{\left||v_{i}^{\prime }-\mu_{j}|\right|}^{2},$$

where $v_{i}^{\prime }$ represents the point of interest motion vector expressed in the 2D annular domain, $\mu_{j}$ is the mean of points in $S_{j}$ and *k* is the maximum number of clusters that we have retained from the observation data. In our experiments we fixed *k* to 10 classes.

Figure 5b illustrates the obtained point of interest assignment to motion classes. As it can be observed, the two persons walking in the same direction, characterized by similar motion patterns, are assigned to the same cluster (marked with blue) as one global object. On the contrary, the points of interest extracted from the pillar present three different motion patterns and are included in independent classes as three different objects. In order to determine the obstacles’ degree of danger, the algorithm needs to identify the various dynamic objects more precisely. To this end, a cluster division/merging procedure based on spatial constraints is proposed.

The quality of the clusters is verified by analyzing the spatial distribution of the points of interest in each class. For each cluster, its associated binary mask (Figure 5c) is constructed as follows. For each point of interest *p*, an associated neighborhood region ${ℛ}_{p}$ is defined, centered on the current point of interest, and with a size twice the grid cell dimensions (cf. Section 3.1.1). The initial clusters are considered as valid if the associated points define connected image regions. Otherwise, the class is divided into multiple groups depending on the number of disconnected regions. On the contrary, if two clusters share in common image regions (i.e., more than 15% of the total binary mask size), the classes are merged together.

Figure 5d presents the object detection results obtained after incorporating into the grouping process the spatial information associated to the points of interest. We can observe that in this case the detected objects are consistent: the two persons walking together are identified as different objects, while the three regions corresponding to different pillar parts are merged into a unique object.

In a general manner, the proposed visual object detection framework proves to be highly effective in identifying fast moving objects. However, the module becomes sensitive in some particular conditions, most notably when: (a) the video camera experiences sudden movements; (b) the video stream is blurred and (c) the object moves on a parallel path with the VI user. In such cases, the motion vectors cannot be estimated efficiently, which penalizes the related detection performances.

On the other hand, large obstructions (e.g., walls or doors) cannot be detected because the object represents more than a half of the video frame size. In such cases, the system cannot distinguish correctly the background from the foreground. In order to overcome such limitations we have considered a complementary obstacle detection module based on ultrasonic sensors. In addition, the sensor information makes it possible to reliably determine the distances to the detected obstacles.

#### 3.1.2. Obstacle Detection Using Sensors Networks

The proposed module is designed to detect and localize the obstacles situated on the walking path of the VI user by using an ultrasonic network architecture properly placed on the waist mounted belt. In order to optimize the sensor distribution on the belt, according to their capabilities of detecting obstructions, we have first evaluated their related performances, with the help of the following three different calibration objects: a cylinder (10 cm diameter and 85 cm the height), a rectangular box (40 cm width and 125 cm height) and a panel (180 cm width and 145 cm height). Such objects are commonly used in the state of the art [10] and are designed to simulate the high variability of instances that a VI user can encounter during navigation. The cylinder can simulate pylons, traffic signs and blocking rods. The box can simulate trees, garbage cans, bumps, pedestrians, while the panel can represent vehicles, walls, fences or bushes.

**Figure 6:**
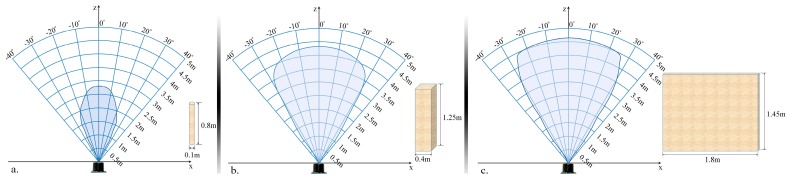
Evaluation of the sensor sonar field of view using: (**a**) a cylinder (10 cm diameter and 85 cm height); (**b**) a box (40 cm width and 125 cm height); (**c**) a panel (180 cm width and 145 cm height).

In order to evaluate the detection range of the ultrasonic sensor, all objects have been moved as follows: (1) from an outside position to the center of the sensors and (2) on the contrary, from the center of the sensors to the opposite outside perimeter. In both cases, an average speed of 1 m/s has been considered. The sensors performance was measured by gradually modifying the distance between the transducer and the considered object from 50 cm to 500 cm in 50 cm steps. For each considered distance, the detectable angles have been estimated. The obtained results are illustrated in Figure 6.

The lowest performances have been obtained for the cylinder (±30 degrees and 350 cm), while the best results are achieved for the panel (±35 degrees and 450 cm). This behavior can be explained by the geometrical structure of the considered objects. From the experimental results presented in Figure 6, it can be observed that regardless of the obstacle size and shape (cylinder, box or panel) the ultrasonic sensor is able to detect, with high confidence scores, objects situated at ±30 degrees and at a distance less than 300 cm.

Such results need to be confronted with the requirements from real-life situations. We have observed that a majority of dangerous obstacles appearing in practice are situated at a distance less than 2 m and at an angle relative to the VI user walking direction ranging between ±50 degrees. This means that the sensor network needs to be designed such that it can ensure coverage of an angle up to 100 degrees.

**Figure 7:**
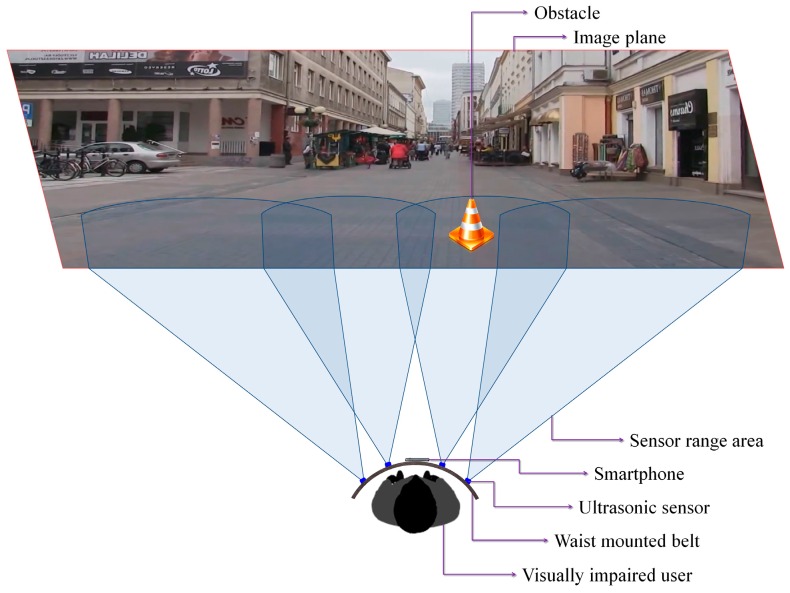
The sensor network distribution and range of action.

Based on these considerations, we have decided to use four ultrasonic sensors properly placed on the waist belt (Figure 7) in order to cover all the area of interest. In addition, to increase the system robustness, the ranging area of each sensor overlaps the adjacent ones. Such a solution offers the best compromise between detection performance, system cost and computational complexity. A lower number of sensors would penalize the performance of the detection module, while a higher number will require more resources while offering no additional benefit over the entire framework performance.

Figure 7 illustrates the sensing abilities of the system and the sonar field of view. Four ultrasonic sensors (MaxSonar LV EZ-0), integrated on a regular belt have been used. They are characterized by the following specifications (cf. the manufacturer’s datasheet): field of view of ±40 degrees and a maximum range of 6 m.

By differentiating between the emitted wave and the measured echo, the microcontroller can estimate the distance between the VI user and the closest obstacle. The sensors are synchronized in order not to interfere. The information is transmitted as a Pulse-Width Modulation (PWM) signal to the receiver. The error estimation is less than 2 cm, which fits perfectly the application constraints. The microcontroller measures the width of the echo pulse and estimates the distances. This information is transferred with the help of the Bluetooth module to the smartphone device. Depending on the sensor that actually performs the detection, the object location within the scene can be estimated as follows: left, right, center-left or center-right of the person.

The smartphone continuously receives information from the sensors through the Bluetooth module and transfers it to the computer vision module. This makes it possible to assign a distance to each motion cluster identified using the foreground detection method (cf. Section 3.1.1). In order to correctly assign distances the video frame is divided into four regions corresponding to the sensors position and field of view (Figure 7).

We have also determined if the object is approaching or is moving away from the VI user by analyzing within successive frames its distance to VI people and the associated motion vectors. An approaching obstacle is marked as *urgent* if is situated in the near vicinity of the user (less than 2 m) otherwise it is marked as a *regular* obstruction. This rough classification helps to better prioritize the acoustic feedback messages depending on their relevance (cf. Section 3.3).

In the particular case when multiple moving objects are present into the same image region we consider that all obstacles are situated at equal distances to the VI person (i.e., the distance indicated by the sensor). Such an approach does not penalize the overall system performance because the warning messages are transmitted only for the closest, *urgent* obstacles.

However, if the sensors indicate the presence of an obstruction in a certain area, this information is automatically transmitted to the acoustic feedback module even if nothing is detected by the computer vision algorithms. On the other hand, fast moving objects with a displacement speed of more than 7 m/s can affect the sensor performance. Therefore, in this case only the vision algorithms can predict the presence of a highly dynamic object in the scene or the existence of an obstruction out of the sensor covering range. For all these obstacles a default distance value of 5 m is assigned to. This value was selected based on the experimental results conducted. The maximum detectable distance of the MaxSonar LV EZ-0 sensor is less than 450 cm, whatever the test object involved. Thus, if an obstacle is identified only by the computer vision module it will be consider as situated outside the sensor action range. In order to keep coherence between the two detection modules, a 5 m distance is assigned for all objects situated outside the ultrasonic reliable detection range.

In order to prioritize the acoustic feedback messages it is important to distinguish between the various obstructions detected and to semantically interpret them. In the following section we introduce a fast method that is able to classify in real-time the detected objects into four major categories corresponding to the most important types of obstructions that a VI user can encounter during navigation: vehicles, bicycles, pedestrians and static obstacles.

### 3.2. Obstacle Recognition Framework

The obstacle recognition framework involves an offline learning process that exploits a training image database structured into four major and generic categories of obstacles: vehicles, bicycles, pedestrians and static obstructions. The categories have been selected in order to correspond to most common types of dynamic/static objects encountered during indoor/outdoor navigation. The class of static obstacles is characterized by a high variability of instances such as: pylons, traffic signs, blocking rod; trees, garbage cans, bumps, walls, fences or bushes. Because the main constraint of our framework is to develop a real-time application we have decided to simplify the classification process to a relatively reduced set of categories (four) rather than perform a generic recognition task (i.e., designed to assign an accurate label to a specific image patch). The learning dataset is composed of 10,000 image patches selected from the PASCAL repository [29]. Each category is characterized by the same number of elements (2500 images). The recognition module receives as input the image patches and performs the following set of steps: relevant interest point extraction, global description of image patches and support vector machine (SVM) training and prediction.

#### 3.2.1. Relevant Point of Interest Extraction

The image patches extracted by the detection modules (cf. Section 3.1) are mapped onto a common format (maximum 12k pixels) while conserving the aspect ratio. Then, for each image a set of points of interest are extracted using the FAST [24] algorithm, and further described using BRIEF descriptors [23]. Even though SIFT [21] or SURF [22] yield high quality features, we have decided to use a FAST points of interest representation because the computational complexity of SIFT or SURF detectors is too high, making them unusable for smartphone-embedded, real-time applications. In comparison, neither SIFT nor SURF can work at full frame rate when integrated on a smartphone device. The FAST extractor operates by considering a circle of 16 pixels around the corner candidate. A pixel is marked as corner if there are *n* contiguous pixels brighter than the point being analyzed.

The extracted points are further described using the BRIEF algorithm that uses binary strings to characterize the informational content of a point of interest neighborhood. The descriptor is highly discriminative, uses a relatively small amount of bits and can be computed based on intensity tests. The differences between two descriptors can be determined by evaluating the Hamming distance (requiring a maximum storage capability of 256 bits), which is much faster than the traditional Euclidian distance.

#### 3.2.2. Global Description of Image Patches

The interest point BRIEF descriptors need to be aggregated into a global image patch representation. One popular approach relay on the Bag of Words (BoW) [30] algorithm, that assigns each interest point descriptor to its closest visual word from a vocabulary. At the end the image is represented as an aggregation histogram. Despite its success, the BoW image representation, relying on large vocabularies, penalize the real-time constraint when integrated on regular smartphone devices because of its memory requirements. In order to overcome such a limitation, we have retained instead a more compact Vector of Locally Aggregated Descriptors (VLAD) representation [31].

The VLAD image description can be regarded as a non-probabilistic variant of Fisher vectors that encode the distribution of interest points according to cluster centers. Compared with BoW VLAD can achieve very competitive retrieval performances with significantly smaller complexity and memory demands (i.e., for an image, VLAD requires 16–256 bytes, while BOW hundreds of Kbytes).

Similar to BoW, but at a reduced scale, a codebook $q:X\to C,C=\left[c_{1},c_{2},\ldots ,c_{K}\right]\in {\mathbb{R}}^{d\times K}$ is learned offline from the training examples (i.e., 10,000 images from the learning database) using the *k*-means clustering algorithm. Here, *C* denotes the set of centroids, while *X* is the descriptor space. So, for each image *I*, *N* points of interest ($I=\left[x_{1},x_{2},\ldots ,x_{N}\right]\in {\mathbb{R}}^{d\times N}$) are extracted using the pyramidal FAST method and each local BRIEF descriptor is assigned to its nearest word in the vocabulary *NN(*x*).* The residual vector *v_i_* can be computed by accumulating all differences between the image descriptors and the associated centroid (i.e., visual word *c_i_*):

(7) $$v_{i}=\underset{x\in I;NN\left(x\right)=c_{i}}{\sum }x-c_{i},\;i\in 1,\ldots ,K$$

To reduce the influence of bursty elements within images the power low normalization has been applied: $v_{i}^{\prime }={\left(v_{i}\right)}^{\alpha }$. The value of α is set to 0.8. Finally, all vectors are *L_2_* normalized in order to have an equal contribution of each cluster in the final vector:

(8) $$v_{i}^{*}=\underset{x\in I;NN\left(x\right)=c_{i}}{\sum }\frac{v_{i}^{\prime }}{{\left||v_{i}^{\prime }|\right|}_{2}},\;i\in 1,\ldots ,K$$

The global image representation using VLAD is given by the concatenation of all the residual vectors $v_{i}^{*}$: $V=\left[v_{1}^{*},v_{2}^{*},\ldots ,v_{K}^{*}\right]$. All vectors $v_{i}^{*}$ have the dimension *d* equal with the size of the local low level descriptor. Therefore, the size of *V* can be determined as *d* × *K*. In the experiments, we have employed a *d* value equal with 256 dimensions for the BRIEF descriptor and a vocabulary size *K* of 256 visual words.

The obtained VLAD descriptor is further normalized to the unit length: $V^{*}=V/\sqrt{K}$. In order to boost up the descriptors we have applied a Principal Component Analysis (PCA) technique [32] that allows adapting the coordinate system to each independent visual word. The PCA reduces the correlation between the various components of the BRIEF descriptor. Hence, at this stage a dimensionality reduction of the descriptors to 128 components has been performed, by saving components corresponding to the highest eigenvalues. Finally, by thresholding the final vector $V^{*}$ we have obtained the binary and zero-centered representation.

#### 3.2.3. Support Vector Machine (SVM) Training and Prediction

The final step of the image recognition module is given by the SVM training (offline process) and SVM prediction (online process). For the offline process, we have used a dataset composed of 10,000 image patches and their associated global VLAD descriptors. We have adopted a SVM classifier with CHI_Square kernel, which has been proven to present powerful generalization capabilities [33]. For each considered category (i.e., vehicle, bicycle, pedestrian and static obstacles) an individual SVM has been trained in order to determine the optimal separation plane between the current class and all other images from the training dataset, while maximizing the margin:

(9) $$f\left(V\right)=\overset{m}{\underset{j=1}{\sum }}y_{j}\cdot \alpha_{j}\cdot CHI\_Square_{kernel}\left(V,V_{j}\right)+b,\;$$

where $CHI\_Square_{kernel}\left(V,V_{j}\right)$ represents the selected CHI-Square kernel; ${\left\{\left(\alpha_{j},y_{j}\right)\right\}}_{j=1}^{m}$ is the training set with $y_{j}\in \left\{-1,+1\right\}$ and b is the hyperplane free term. For each category, a single SVM classifier is trained using one-versus all strategy (i.e., the images from the current class are considered as positive examples while all other images from the training dataset are used as negatives samples. The SVM model creates a partition of the 128 dimensional VLAD feature space into separate categories.

In the online phase, for each image patch extracted by the obstacle detection module, a global representation using binary VLAD descriptors is constructed. This representation is applied as input to the trained SVM models. The one-versus-all classification method has been used in order to predict the image patch category. A patch is tested against all trained SVM and the one with the greatest margin and f(V)>0  is selected as representative.

To satisfy the real-time constraint imposed to the application, the entire process has been parallelized on multiple threads. The number of threads is adaptively adjusted depending on the total number of objects detected in the scene. In addition, in order to deal with the situation when an image patch cannot be confidently assigned to any of the considered classes (i.e., the output of SVM is negative f(V)<0) we have introduced an extra category denoted Outlier.

The overall performance of the obstacle classification method is influenced by the size and the content of the training image dataset used in the offline learning process. We can expect that a higher number of training examples can lead to a better estimation of the separation hyperplanes. Still, increasing the size of the learning data set is not sufficient. In order to avoid overfitting problems [33], a relatively high variability of appearances need to be ensured. In our case, because the main constraint is to develop a real-time application, we have considered a reduced set of categories (four), with 2500 images per class. As the experimental evaluation (cf. Section 4) shows, this is sufficient to ensure high recognition rates.

### 3.3. Accoustic Feedback

The acoustic feedback is responsible of informing the VI user about the presence of different types of obstacles, either static or dynamic. The main goal of the proposed system is to transmit warning messages fast enough, so that the VI person can walk normally while avoiding dangerous situations. At the hardware level, we propose using Bluetooth bone conduction headphones. In this way, the user’s ears are not covered and the user can continue to capture the other external sounds from the surrounding environment.

After discussions with two associations for the blind we have decided that it is better suited to use acoustic alerts rather than tactile stimulation. Most of the VI persons questioned consider that vibrations are insufficient to capture the overall dynamics of the environment. Furthermore, systems adopting tactile simulation are considered as invasive because they require an actual physical contact with the human skin. The detection of the obstacle distribution within the scene, the semantic interpretation of the identified objects and the effective transmission of this information to a VI user are the key elements for an effective usage of any ETA. However, the manner of transmitting the acoustic feedback plays a central role in the acceptance of the device by the users.

The proposed framework uses voice messages rather than beeping. The beeping strategy can only warn users about the proximity of an obstacle and not about the type of obstruction or relative position of it. As an example, the VI user is informed about the presence of an approaching vehicle situated in his/her near surrounding as “*urgent* vehicle”, which makes the acoustic framework highly intuitive without need of any particular training.

**Figure 8:**
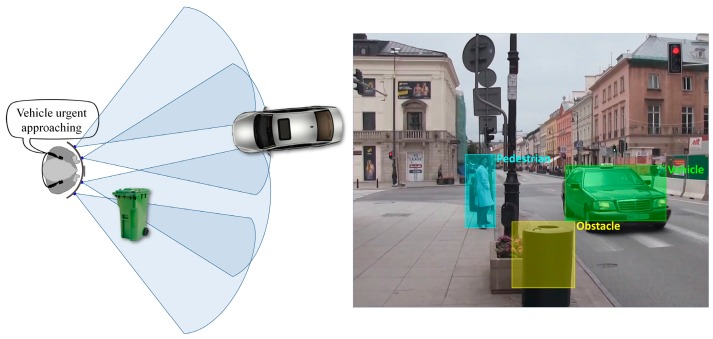
Warning message prioritization based on the object semantic interpretation.

In order to avoid confusing the VI user with too much information, the acoustic signals are transmitted with a minimum refresh rate of 2 s. The classified objects are analyzed and prioritized depending on their potential level of danger. For the situation presented in Figure 8, an acoustic warning saying “*urgent* vehicle” is sent to the VI user, rather than a message announcing the presence of an obstacle in the near vicinity.

Table 2 presents the set of warnings considered by the system and their associated order of relevance (i.e., 1 representing the element with the highest importance). After applying the object recognition method (cf. Section 3.2) the system can semantically interpret the detected obstacles based on their degree of danger. In this context, an alert message about a vehicle situated at a distance superior to 2 m relative to VI user (*regular* obstruction) will be transmitted before a warning regarding a pedestrian situated in the *urgent* area. After the discussions with the association for the blind involved in the project it was decided to give higher importance to approaching vehicles based on their speed of displacement and degree of danger. A possible collision with a fast approaching vehicle will have a higher impact over the VI user when compared to a possible collision with a walking pedestrian or an obstruction.

In addition, in order to infer information about the object location within the considered environment, the set of acoustic signals is stereo-encoded. Thus, if the detected object is situated on the left side of the VI user, the warning message will be transmitted on the left helmet of the bone conducting headphones and vice-versa for obstructions located on the right side of VI person. For obstacles identified in front of the VI, the alarms are transmitted in both helmets. For far away objects situated at a distance more than 3 m, a higher confidence is given to the vision module, while for closer objects localized at a distances less than 3 m a higher confidence is given to the sensor module.

## 4. Experimental Results

In order to validate the proposed approach, an extensive objective evaluation of the system, presented in the following section, has been performed.

### 4.1. Objective Evaluation

**Figure 9:**
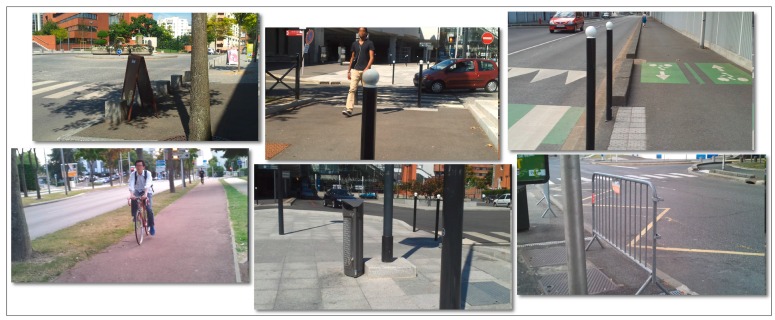
Examples of the videos used in the experimental evaluation.

The proposed assistive device has been tested mostly in outdoor environments because of the high complexity and diversity of static/dynamic objects. Regarding the computer vision-based obstacle detection module the video camera embedded on the smartphone has been used as an acquisition device. The video stream is acquired at a frame rate higher than 25 images with a resolution of 320 × 240 pixels. A testing database has been developed, including 20 items with the average duration of 10 min. Figure 9 shows some examples of the recorded videos. Even though the video camera is mounted on VI user waist belt, the captured video streams can exhibit various types of motion, sudden changes in the lighting intensity or contain trembled and cluttered images.

When a ground truth data set is available, the obstacle detection/classification errors can be globally described with the help of two error parameters, denoted by $N_{MD\left(MC\right)}$ and $N_{FD\left(FC\right)}$, and respectively representing the number of missed detected (resp. missed classified) objects and the number of falsely detected (resp. false classified) obstacles. Let us denote by $N_{D\left(C\right)}$ the total number of correctly detected (resp. classified) obstacles.

Based on these entities, the object detection/classification performances can be globally evaluated using the precision (*P*), recall (*R*) and *F*-norm (*F*) parameters, extensively used in the state of the art for evaluation purposes [34] and defined as:

(10) $$P_{D\left(C\right)}=\frac{N_{D\left(C\right)}}{N_{D\left(C\right)}+N_{FD\left(FC\right)}},\;R_{D\left(C\right)}=\frac{N_{D\left(C\right)}}{N_{D/C}+N_{MD\left(MC\right)}},\;F_{D\left(C\right)}=\frac{2\cdot P_{D\left(C\right)}\cdot R_{D\left(C\right)}}{P_{D\left(C\right)}+R_{D\left(C\right)}};$$

The precision *P* is a measure of fidelity and is inversely related to the first type of errors (false positives). The recall measure can be interpreted as a measure of completeness, being inversely related to the second type errors (false negatives). An ideal system should achieve both precision and recall scores close to 1.0. The recall and precision values can be combined into a single measure denoted by *F* (or *F-score)*, which helps to determine the overall module performance, by considering as equally important the precision and recall scores.

Table 3, Table 4 and Table 5 present the results obtained for the obstacle detection module. Table 3 presents the detection performance achieved when activating only the computer vision module. Table 4 gathers the detection rates of the sensors module, while in Table 5 the overall system performance is evaluated. All the experiments have been performed in real life without any simulated environments. The difficulty in the evaluation is given by the lack of reproducibility of the outdoor conditions. Even though, for the vision module we can record the video stream and then perform the detection to determine the system performance. In the case of the ultrasonic module such an approach is intractable. So, all the experimental results were obtained by actual VI users walking in real outdoor environments.

**Figure 10:**
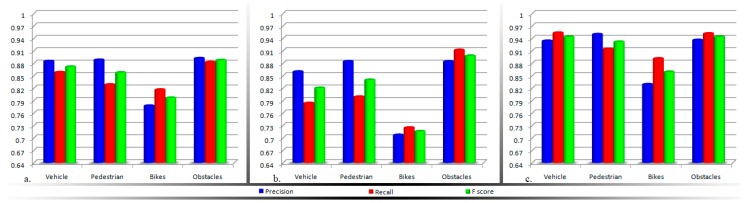
Experimental evaluation of the obstacle detection module, when activating: (**a**) The computer vision system; (**b**) The sensor network; (**c**) The overall framework.

For better visualization, the information gathered in Table 3, Table 4 and Table 5 are synthesized in Figure 10.

**Figure 11:**
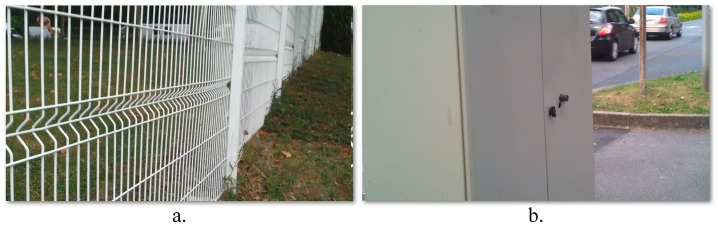
Detected objects missed: (**a**) By the ultrasonic sensor system; (**b**) By the computer vision system.

Based on the analysis of the experimental results obtained, the following conclusions can be highlighted:

1. The computer vision techniques return higher detection scores when compared with the ultrasonic approach. This behavior can be explained on one hand by the sensor sensitivity to highly dynamic objects (e.g., vehicles or bicycles) or hollow objects. Figure 11a illustrates an obstruction for which the sensor sensitivity is high, because of the object’s geometrical structure (the fence is characterized by a large number of hallows). In this case, no echo signal is received. On the other hand, because the vision algorithms track an object between multiple frames, the system is able to reduce the number of false alarms and the accidental missed detections.
2. The field of view of a regular video camera is superior when compared to the sonar range of action. As a consequence, the vision algorithms are able to detect the faster moving obstacles situated at longer distances (ranging from ten to twenty meters). For sonars, in the ideal case, the detection distance is limited to five meters.
3. As expected, the vision methods are highly sensitive to the quality of the estimated motion vectors. Because the static obstacles are identified based on the camera apparent motion analysis, if the user stops suddenly, the system losses the motion information and cannot identify static obstructions. For large objects, that cover more than a half of the image size, the system returns a high number of missed detections. On the other hand, the sensor module proves to be highly robust to all the above situations. Figure 11b illustrates an obstruction frequently encountered during outdoor navigation. Because the object size covers more than a half of the video frame, a high number of interest points is extracted from this foreground object in the detriment of all the background elements. After applying the RANSAC algorithm the objects’ key-points will be marked as *inliers* and will be considered as part of the background/camera motion, while the *outlier* interest points (actually belonging to the background elements) will contribute to the subsequent clustering process and will be interpreted as various obstacles existent in the scene.
4. When the two detection modules are put to work in common, an important improvement is observed, with more than 6% on the *F-score*, regardless of the obstruction type or nature (i.e., static or dynamic).

In the second part, we have focused on the evaluation of the obstacle classification module that allows the semantic interpretation of the detected obstacles. The recognition system receives as input the image patches extracted using the vision-based object detection module. Table 6 presents the confusion matrix for each of the considered categories. The number of elements in the considered ground truth (GT) is also indicated here.

As mentioned in Section 3.2 we introduced an extra category, called “Outlier”. When an image patch cannot be confidently assigned to any of the considered classes, it will be marked as outlier. The proposed strategy makes it possible to assign an image to a category based on its resemblance to the training dataset and not just because it has to belong to a given class.

Concerning the acoustic feedback, for all image patches classified as Outliers only a BEEP warning is sent to the VI user. For objects identified only by the sensor module it is impossible to assign semantic information. In this case, in order to inform user about the presence of a possible dangerous obstruction a BEEP warning is sent into the bone conduction headphones.

The experimental evaluation of the recognition module was conducted on a set of 1190 image patches. The classification-related precision, recall and *F-score* measures are presented in Table 7.

The experimental results obtained validate the proposed approach, with a classification *F-score* superior to 82% for each of the considered categories. The lowest rates are obtained for the bicycle class. This behavior can be explained by the images used in the training phase. As indicated in Section 3.2, we have used to develop the training dataset images from the PASCAL repository, which includes images of static bicycles without any humans. Because the detection module is based on motion information analysis, for bicycles with people riding them both objects are characterized by the same motion pattern. So, the system is not able to distinguish between them and extracts as output only one image patch that contains both objects as a whole. However, for all other categories the performance of the module is far better than an average of 90% results.

Regarding the computational complexity, the average processing time of the proposed framework when activating all modules (obstacle detection based on computer vision algorithm, sensors networks and object recognition) is around 100 ms, which leads to an average processing speed of 10 frames per second. The experimental results were performed on a regular Android smartphone device (Samsung S7). In terms of the battery consumption, the smartphone and the sensor module are simultaneously powered from an external battery of 16,000 mAh that allows running our system continuously for 5 h.

### 4.2. Subjective System Evaluation

In order to determine the performance of the proposed framework we have used for evaluation a set of 21 visually impaired people with ages ranging between 27 and 67 years. The main goal of testing was to gather information related to the following aspects: are the users able to start the application by their own? Can they safely navigate in a novel environment? Is it possible to avoid obstacles using the set of acoustic warnings? Is the system globally useful and can it complement the white cane?

The testing was performed in various urban scenes for which VI people have no initial knowledge about the objects location, position or size. The proposed system was evaluated in various arbitrary locations and not under a specific environment. Each VI person was asked to walk on a test path and to use both the proposed device and the white cane. After a participant completed the task, an observer conducted a post-test debrief interview with users about the behaviors they observed during the testing. After discussions with the participants, the following conclusions can be highlighted:

1. At the beginning of the testing phase the older VI people prefer to rely on other senses in order to complete the navigation scenario rather than listening to system acoustic signals. We have observed retention and mistrust to innovations. However, in the case of young VI users the system had a great impact and they expressed the willingness to use our framework frequently in daily activities.
2. After an initial training phase, performed with the help of technicians or carriers, most participants expressed that the system is useful and easy to learn. Even the people without sufficient abilities to handle a smartphone device expressed strong interest in the proposed architecture.
3. VI persons found the system wearable, friendly, light weighted and non-intrusive satisfying the hands-free and ears-free requirements. Even the older VI people consider the proposed system light weighted because the weight of all components summed together is inferior to 750 grams (i.e., the regular smartphone device (Samsung S7)-152 grams, Bluetooth bone conducting headphones (AfterShokz Bluez 2)-41 grams, the microcontroller (Arduino Micro)-6.5 grams, the bluetooth device (Master Slave HC-05)-10 grams, 4 ultrasonic sensors (MaxSonar LV EZ-0)-4.23 grams, an external battery (Anker Astro E5 16,000 mAh)-308 grams and a Smartphone waist belt-200 grams).
4. The various functions included in the architecture were well integrated and the warning messages are transmitted fast enough so users can walk normally.
5. The usage of bone conducting headphones is appropriate because they allow hearing other sounds from the environment. The speech feedback is considered better than any vibro-tactile stimulation or an acoustic strategy based on beep patterns. The beeps can warn user about the proximity of an obstacle and not about the relative position or degree of danger. Moreover, inferring additional cognition over the scene is impossible.
6. Most participants pointed out that the system is very useful when combined with the white cane, but they do not feel confident enough to use it as a standalone device. The white cane is designed to detect possible obstructions situated in the near vicinity of the user, but it requires an actual physical contact with the object. However, it is not able to provide additional information elements such as: the speed and type of object the user is encountering, the static or dynamic nature of the obstacle, the distance and time to collision. Even so, the white cane is the cheapest, the simplest and the most reliable element used as navigation aid. Because, the VI people are very familiar with the white cane they feel reticence and uncertain to completely switch to this assistive device, so any novel electronic travel aid (ETA) should be designed not to replace the cane, but to complement it in an intelligent manner, with additional functionalities. This is also the purpose of the proposed system.
7. Users were satisfied with the acoustic feedback even when tested in crowded outdoor scenes with multiple moving objects and obstructions. Moreover, VI people were enthusiastic when they realized that the system is able to detect obstacles situated at head level.

## 5. Conclusions and Perspectives

This paper has introduced a novel electronic travel aid that facilitates the safe navigation of VI people in urban scenes by using both sensors and computer vision technologies. At the hardware level, the proposed system is composed of a regular smartphone device, ultrasonic sensors, Bluetooth module, microcontroller, bone conduction headphones and a waist belt. At the software level, the major innovation of the proposed assistive device concerns the fusion of two completely independent sources of information: ultrasonic sensors and video streams. The system can identify with high confidence static or highly dynamic objects existent in the scene regardless on the object location, size or shape. In addition, in contrast with the most of the state of the art systems, the proposed prototype is able to semantically interpret the detected objects and to establish their degree of danger. The visually impaired user is informed by the presence of different obstacles in his/her near vicinity through a set of acoustic signals.

To demonstrate the reliability and robustness of the proposed architecture we have performed an extensive experimental evaluation with actual VI people. First, we have provided an objective evaluation of the obstacle detection and classification modules, while in the second part we have focused our attention in introducing a subjective evaluation of our system and we have presented the VI people degree of satisfaction and comments after using our prototype. The subjects found our system friendly, lightweight, wearable and non-intrusive, satisfying both the hands-free and ears-free requirements. Moreover, the computational time is reduced and the warning messages are transmitted fast enough so that the VI user can walk normally.

For future work we plan to extend our framework with additional functionalities such as: providing navigational information so that a VI user can reach a desired destination, the development of a personal shopping assistant, or an additional face recognition capability to allow the identification of familiar faces. Moreover, it is necessary to derive a deeper level of understanding of the surrounding scene in order to provide additional cognition over the environment.

## Figures and Tables

**Table 1:** Evaluation of the assistive device systems dedicated to visually impaired people.

		System	Cost	Real-Time	Portable	Wearable	Reliable	Friendly
Sensor-based ETA	-	Mowat sensors [3]	-	√	√	-	-	-
		Trisensor [4]	high	√	√	-	√	-
		Kuc [5]	moderate	√	√	√	√	-
		GuideCane [6]	high	√	-	-	√	-
		EPFL [7]	moderate	√	√	√	-	-
		CyARM [8]	high	√	√	-	-	√
		SUGAR [9]	low	√	√	-	-	√
		Jeong et al. [10]	high	√	√	-	-	-
Computer Vision based ETA	CCD video camera	SmartVision [11]	low	-	√	√	-	√
		Manduchi et al. [12]	low	√	√	-	-	√
		Tapu et al. [13]	low	√	√	√	-	√
	3D cameras	Saez et al. [14]	high	-	√	√	-	√
		Pradeep et al. [15]	high	-	√	√	√	-
		Sáez et al. [16]	low	√	√	√	-	√
		Khan et al. [17]	moderate	√	√	√	-	-
		Takizawa et al. [18]	high	-	√	√	-	-
		Brock et al. [19]	moderate	√	√	√	-	√
		Panteleris et al. [20]	low	-	√	√	-	√

**Table 2:** The set of acoustic warning messages.

Order of Relevance	Object Type	Distance to the VI User ^1,2^	Sound Pattern
1	vehicle	*urgent*	Urgent vehicle
2	bicycle	*urgent*	Urgent bicycle
3	vehicle	*normal*	Normal vehicle
4	pedestrian	*urgent*	Urgent people
5	obstruction	*urgent*	Urgent obstruction
6	bicycle	*normal*	Normal bicycle
7	pedestrian	*normal*	Normal people
8	obstruction	*normal*	Normal obstruction

^1^ An object is marked as *urgent* if its associated distance relative to a VI user is inferior to 2 m, otherwise is considered as normal; ^2^ An object is considered as approaching the subject if the associated distance between successive frames is decreasing, otherwise is marked as departing the VI person.

**Table 3:** Experimental evaluation of the obstacle detection module using computer vision techniques.

Category	GT ^1^	N_D_	N_MD_	N_FD_	P_D_	R_D_	F_D_
Vehicle	431	370	61	48	0.88	0.85	0.87
Bicycle	120	98	22	34	0.77	0.81	0.79
Pedestrian	374	310	64	39	0.88	0.82	0.85
Obstructions	478	412	66	51	0.88	0.86	0.87

^1^ GT—represents the total number of objects in the ground truth dataset.

**Table 4:** Experimental evaluation of the obstacle detection module using sensors networks.

Category	GT	N_D_	N_MD_	N_FD_	P_D_	R_D_	F_D_
Vehicle	431	338	93	55	0.86	0.78	0.82
Bicycle	120	87	33	36	0.71	0.72	0.72
Pedestrian	374	299	75	39	0.88	0.79	0.83
Obstructions	478	436	42	57	0.88	0.91	0.89

**Table 5:** Experimental evaluation of the overall obstacle detection architecture.

Category	GT	N_D_	N_MD_	N_FD_	P_D_	R_D_	F_D_
Vehicle	431	411	20	29	0.93	0.95	0.94
Bicycle	120	107	13	22	0.82	0.89	0.85
Pedestrian	374	341	32	18	0.95	0.91	0.93
Obstructions	478	455	23	31	0.93	0.95	0.94

**Table 6:** Confusion matrix between the considered categories.

	Vehicle	Bicycle	Pedestrian	Obstructions	Outliers	GT
Vehicle	347	4	0	4	15	370
Bicycle	3	86	7	0	2	98
Pedestrian	0	18	278	8	6	310
Obstructions	4	3	12	378	15	412

**Table 7:** Experimental evaluation of the obstacle classification module.

Category	GT	N_C_	N_MC_	N_FC_	P_C_	R_C_	F_C_
Vehicle	370	347	23	7	0.98	0.93	0.95
Bicycle	98	86	12	25	0.77	0.87	0.82
Pedestrian	310	278	32	19	0.93	0.89	0.91
Obstructions	412	378	34	12	0.96	0.91	0.94
